# Voices in methodology: analyzing self-mention markers in English and Persian psychology research articles

**DOI:** 10.3389/frma.2024.1336190

**Published:** 2024-04-17

**Authors:** Fatemeh Moradi, Mohammad Reza Montazeri

**Affiliations:** Department of English Language and Literature, Allameh Tabataba'i University, Tehran, Iran

**Keywords:** academic writing, authorial identity, disciplinary differences, method section, self-mention markers

## Abstract

Although previous preconceived notions discourage authors from asserting their presence in research articles (RAs), recent studies have substantiated that the use of self-mention markers offer a means to establish authorial identity and recognition in a given discipline. Few studies, however, explored specific sections of research articles to uncover how self-mentions function within each section's conventions. Exploring the use of self-mention markers, the present study aimed at comparing the method sections written by native English writers and L-1 Persian writers in the field of psychology. The corpus contained 120 RAs, with each sub-corpora including 60 RAs. The RAs were then examined structurally and functionally. The data were analyzed both quantitatively, using frequency counts and chi-square analyses, and qualitatively through content analysis. The findings indicated a significant difference between English and Persian authors concerning the frequency of self-mentions and the dimension of rhetorical functions; however, the differences in the dimensions of grammatical forms and hedging and boosting were found insignificant. Native English authors were inclined to make more use of self-mentions in their research articles. The findings of the current study can assist EAP and ESP novice researchers in taking cognizance of the conventions of authorial identity in each genre.

## 1 Introduction

Over the past few decades, there has been a notable shift in the acceptance of author visibility in academic writing through using explicit self-reference in academic writing. Researchers are frequently confronted with decisions regarding the degree of visibility or neutrality in their research articles (RAs) (Can and Cangir, 2019). On the one hand, there exists a desire among researchers to maintain a neutral and unobtrusive presence within the RA (Webb, 1992). On the other hand, they may opt to establish a clear authorial presence to engage with readers. Corpus-based studies (e.g., Kuo, 1999; Harwood, 2005; Sheldon, 2009; Can and Cangir, 2019; Walková, 2019; Deng and He, 2023; Dontcheva-Navratilova, 2023) have unveiled the diverse functions of these self-references in successful academic writing (Hyland, 2002a), from structural organization, expressing personal opinions and acknowledging claims, to establishing relationships with readers and their discourse community.

Strategic use of self-mention markers allows scholars to craft an authoritative voice that positions them as adept and dependable members of their academic community (Mur Dueñas, 2007). Furthermore, as argued by Mur Dueñas (2007), providing readers with a sense of research validity and relevance requires the construction of a trustworthy ethos. The application of self-mentions can be one way of achieving this level of credibility. By employing these markers, researchers signal their active involvement and expertise in the subject matter, thus bolstering the overall trustworthiness of their work. Underscoring the strategic role of self-mentions in academic discourse, Hyland (2004a) maintained that self-mentions serve to establish one's credibility and expertise, aligning with a specific viewpoint to gain recognition for individual perspectives and research choices.

Previous studies have maintained that genre-related features (Swales, 1990; Bunton, 1998; Paltridge and Starfield, 2007), the intended audience (Shaw, 2000), and cultural differences (Sanderson, 2008; Mauranen et al., 2010) are the main three factors that influence authors' rhetorical inclination indicating authorial presence in academic texts. Editorial corrections and referee reports are ways of putting the seal on research articles in order to adhere to the expectations of the target community (Can and Cangir, 2019). Hence, writing guidelines and audience expectations may be the causal agents of discrepancy in stance and voice of academic writings.

Honoring the readers' expectations and beliefs and manifesting indivisibility with a particular community members are not the sole techniques for having an efficacious writing. Showing innovation and relevance are also of great significance in this regard (Myers, 1989), and the use of self-mention markers is effectual in expressing innovative ideas and novelty (Hyland, 2001). Thus, referring explicitly to one's own role as a writer have “significant consequences for how one's message is received” (Hyland, 2001, p. 211). As a result, enhancing the researcher's status and the research itself are the main outcomes of establishing an authorial presence; furthermore, it affects the relationship between the author and readers and the way former is deemed by the latter (Mur Dueñas, 2007).

According to Martínez (2004), international English-speaking community tend to make use of more first person pronouns in order to improve the status of their claims. Moreover, it has been suggested that impersonality should not be rendered as a defining characteristic of academic writing, and the writers' presence need to be established according to the context (Ivanic, 1998; Kuo, 1999; Tang and John, 1999; Hyland, 2001).

Influenced by their cultural background, Persian authors tend to adopt an impersonal writing style, showcasing humility and avoiding the use of first-person pronouns (Zarei and Saadabadi Motlagh, 2019). The choice of personalization level and the use of self-mentions pose challenges for non-native writers, particularly Persian authors, for various reasons. Firstly, authorial identity varies across specific disciplines and discourses, each having its preferred personalization patterns (Hyland, 2001; Fløttum et al., 2006). Other reasons why non-native writers, especially Persian writers, find it difficult or tend to avoid using self-mentions arise from their cultural and epistemological background (Dontcheva-Navratilova, 2023), academic writing norms (Zarei and Saadabadi Motlagh, 2019), and previous learning experiences (Lee and Deakin, 2016). Given the scarcity of studies examining the frequency, role, and functionality of identity in academic papers written by Iranians, the current study aspires to establish foundational insights into the writing practices of Iranian authors in the field of psychology.

## 2 Review of literature

A multitude of studies have investigated the differences between academic writing style of Anglophone and non-Anglophone communities (Berkenkotter and Huckin, 1995; Canagarajah, 1996; Flowerdew, 1999; Curry and Lillis, 2004; Tardy, 2004). One large-scale, corpus-driven study conducted by Hyland (2001) examined the authorial presence by the use of personal pronouns in 240 RAs from eight various disciplines. The results indicated a conspicuous difference in the utilization of self-mention markers between hard (e.g. engineering) and soft (e.g. sociology) science texts. More personal pronouns were applied by the researchers writing in the field of soft sciences (about 30 examples in each paper) than those written by researchers in a hard science field (about 10 examples in each paper). Soft science researchers were inclined to use the pronoun “I”, while the use of “We” was recurrent in hard science papers. In another study, Hyland (2002b) compared academic texts written by student writers and expert writers. The results showcased that the use of personal pronouns by expert writers was four times more than the student writers who were predisposed to exploit passivization for presenting and discussing their arguments.

Mur Dueñas (2007) analyzed the use and distribution of self-mention markers and self-citations comparatively between a corpus of business management RAs written in English and Spanish. It was revealed that English writers made more use of self-mentions than the Spanish writers. The different results in the sub-corpora implied the fact that the use of self-mention markers is not merely dependent on the authors' discipline, but the cultural context affect the use of self-mentions in RAs as well.

With a focus on first person function and distribution, Martínez's (2004) study attempted to investigate the similarities and differences in the utilization of first person in different sections of research articles written in the field of biology by native and non-native English writers. Phraseological problems, overuse, and underuse were discovered in the non-native English speaking corpus. Native English writers made more use of first person in the result section mainly due to the fact that they felt responsible about the methodological decisions that led to the results. The study also directed attention toward the fact that raising non-native English writers' awareness regarding the use of first person in various sections of RAs is of great significance.

Taking a linguistic approach, Breivega et al. (2002) presented a pilot study which investigated the authorial presence and stance in RAs from three different disciplines of medicine, economics and linguistics in three languages of English, French and Norwegian. Another study conducted by Vassileva (1998) attempted to establish specific cross-cultural variation in the utilization of “I” vs. “we” in academic discourse between five languages of English, German, French, Russian, and Bulgarian. Data for the study included 5 corpora of randomly selected articles. The results indicated that native English writers made use of “I” more than the other groups indicating a greater author presence in English corpus.

Sheldon (2009) delved into different identities behind first-person roles in English and Spanish. The corpus for the study included 18 English and 18 Spanish RAs in Applied Linguistics and Language Teaching. Commonalities and differences were identified in the distribution of first-person pronoun in the articles, and the results revealed that the authors in both languages inhibit the text in different ways which implied the fact that the construction of self-representation differs across written cultures.

Molino's (2010) study attempted to decide on the impressionability of personal and impersonal authorial references to variation across academic writing cultures. The study tried to analyze the utilization of first-person pronouns in Linguistics research articles in English and Italian cross-culturally. Differences in the frequency of use of personal and impersonal authorial references were identified. Picking different interpersonal strategies, objectivity or subjectivity in the two academic discourse communities appeared to be the main reasons of these disparities. Furthermore, Karahan (2013), in a small-scale study, investigated the frequency and distribution of personal pronouns of “I” and “WE” in 20 research articles written by Turkish authors and 20 research articles written by non-Turkish writers in English. The results indicated that both groups of authors were inclined to use more “WE” than “I” in their academic writings. Passive structures and other depersonalization strategies were more frequent in the Turkish corpus.

In 2018, Can and Yuvayapan (2018) analyzed how Anglophone and Turkish PhD students use interactional metadiscourse in stance-making. The study focused on identifying the types, frequency, and variety of these markers in their dissertations. Data from 120 soft science dissertations showed that native English speakers used more self-mention markers, while non-native writers used “we” more often, possibly due to cultural differences. In a related study, Can and Cangir (2019) compared self-mention markers in dissertations of Turkish and British doctoral students in literary studies. Their results revealed significant differences, with British authors using “I” more frequently. The surrounding context of these markers also showed divergence. In another recent contrastive study, introducing a three-dimensional model for understanding self-mention in academic writing, Walková (2019) analyzed self-mention markers produced by in L1 English, L1 Slovak, and L2 English by Slovak authors. She found that the use of self-mention in L2 English academic writing by Slovak linguists differed from L1 Slovak in terms of frequency, grammatical forms, and levels of hedging/boosting. The only similarity lied in the rhetorical functions of self-mention, suggesting that self-mention practices may not uniformly transfer from one's native language to L2 English.

In a more recent study, Chen (2020) explored self-reference and its influence on the development of pragmatic competence in a report on a series of survey studies analyzing Chinese writers' Academic Written English (AWE). He found that undergraduate theses closely resembled AWE conventions, while master's theses moved toward Academic Written Chinese (AWC) conventions. Doctoral dissertations and conference abstracts, on the other hand, tended to revert to AWE conventions but remain distant. This study underscores the influence of identity and social values on academic discourse, illustrating how Chinese AWE writers manage to assert authority while also projecting modesty, shaped by their cultural background. In another study, Deng and He (2023) also compared stance markers in English and Chinese research article conclusions across soft and hard sciences. Analyzing 20 years of data from 180 conclusions per language in four disciplines, they identified cultural and disciplinary differences in academic writing conventions. Based on the results, they found that English and soft science writers tended to use hedges and self-mentions more frequently, while Chinese and hard science writers favored boosters and attitude markers.

In an attempt to explore self-mention markers in the context of Czech master's theses written in English, Dontcheva-Navratilova (2023) analyzed three dimensions of realization, authorial roles, and distribution across rhetorical sections in the field of humanities. Having compared three corpora of Czech theses with L1 learners' and published academic texts, Dontcheva-Navratilova found that Czech graduates tended to display lower authority and favored humility, diverging from disciplinary norms. She concluded that this tendency was influenced by an amalgam of L1 and L2 academic conventions, lower rhetorical maturity, and the context of master's thesis.

As to the significance of the use of self-mention markers, it was found that it constructs the writer's stance toward the ideas being expressed (Mur Dueñas, 2007); moreover, they can be regarded as metadiscursive features. A category in Hyland (2004b; 2005) interactional metadiscourse and Hyland and Tse's (2004) taxonomies is allocated to self-mention markers. Hyland's (2005) model of interactional metadiscourse markers accounted for the extent to which the author attempts to co-construct a text with readers. A previous taxonomy proposed by Hyland (1999, 2000) included self-mention markers as a category of interpersonal metadiscourse which was referred to as “person markers.” Their relation with other metadiscourse categories have been addressed, but self-mention markers have not been regarded as a dependent category in other taxonomies (e.g., Crismore et al., 1993; Dafouz Milne, 2003).

As crucial features of writer identity, rhetorical self-projection through the use of specific linguistic forms, such as first person pronouns, are exploited to showcase the authorial power in a given discourse (Tang and John, 1999; Albalat-Mascarell and Carrió-Pastor, 2019). Studies examining the use of first person in academic essays have found that writers can convey different levels of authority through their writing, depending on how they choose to present themselves in the discourse. This suggests that the use of the first person in academic writing is inextricably linked to the writer's identity and can influence how their work is perceived (Tang and John, 1999).

Based on Halliday's (1994; 1998) Systemic Functional Linguistics, language is not a manifestation of reality, but it, *per se*, “creates” the reality. Given the significance of “self” and authorial voice through writing, Tang and John (1999) proposed a taxonomy for categorizing how first person pronouns (including *I, me, my, mine, we, us, our*, and *ours*) reveal the identity of an author within the genre of academic writing. Their taxonomy classifies authorial identities into six genre roles: “the representative”, “the article guide”, “the article architect”, “the research process recounter”, “the opinion-holder”, and “the originator” (p. 27–29).

Chávez Muñoz (2013) expanded on Tang and John's (1999) by introducing a new category, 'I' as the interpreter, positioning it between the roles of opinion-holder and originator. In addition, Hyland (2002a) focused on pronouns exclusive to the reader and provides a taxonomy encompassing “expressing self-benefits”, “stating a purpose”, “explaining a procedure”, “elaborating an argument”, and “stating results/claims”. Another category is that of Mur Dueñas (2007) who identified eight rhetorical functions of exclusive 'we', including delineating the goal or purpose of the study, outlining the steps followed in the research, explaining procedures employed, stating hypotheses or expectations, evaluating research limitations, assessing the strengths of the study, making statements or claims to support arguments, and presenting results and findings.

Comparing these taxonomies, Walková (2019) proposed a fresh taxonomy outlined in Table 1, which covers the substantial overlap among other taxonomies. Walková's (2019) taxonomy encompasses a range of rhetorical functions, corresponding grammatical forms, and levels of hedging and boosting, providing a comprehensive framework for understanding authorial identity in academic writing.

**Table 1:** Three dimensions of self-mention in academic writing.

**Rhetorical function**	1) Stating contribution 2) elaborating argument, presenting opinion, stating knowledge 3) describing or explaining research decision or procedure 4) stating purpose, intention, or focus 5) acknowledging others
**Grammatical forms**	1) Subjective pronouns 2) Possessive Pronouns 3) 3. Other
**Hedging and boosting**	1) Boosting 2) Hedging 3) Neutral

Self-representation in academic writing has been spotlighted by researchers over the past decades. However, few researchers have sought to comparatively examine the utilization and functionality of self-mention markers by native and non-native authors within a specific discourse or genre, particularly in a single section of research article. Moreover, compared to the introduction and discussion sections, methods section structure has received less attention from researchers except for cases in which the whole article is under focus. To this end, the present study aims to contrastively analyze the use of self-mention markers in the method section of psychology research articles written by native English and Persian writers. Based on the research purposes, the following research questions are raised in this study.

- Is there any difference in the frequency of self-mention markers in the method section of psychology research articles written by native English and Persian writers?
- What are the differences, if any, between English writers and Persian writers in terms of the functionality of self-mention markers in the method section of the research articles?

## 3 Corpus and methodology

The data for the comparative analysis includes the methodology sections from 60 psychology research articles written by Persian writers and methodology sections from 60 psychology research articles written by native English writers. The methodology sections of the research articles were randomly selected from different prestigious journals published between 2015 and 2023. We gleaned the English Corpus (EC) from the following journals: *Cognition, Journal of memory and language, Cognitive psychology, Brain, behavior and immunity*, and *Journal of personality*. Additionally, we collected the Persian Corpus (PC) sample from the following journals: *Iranian rehabilitation journal, Iranian journal of clinical psychology, Iranian Journal of Social Sciences and Humanities Research, International Journal of high risk behaviors and addiction*, and *Iranian journal of psychiatry and behavioral sciences*.

The research papers were selected randomly from among the articles written each year. We also sought to opt for only English and Persian writers and double-checked the co-authors' first languages as well. As for the selection of each article based on this criterion, we followed Wood's (2001) definition of an L1-English writer. That is, we primarily checked their affiliation with either English-speaking or Persian-speaking institutions and then ensured their first name and surname native to either of these languages. We even excluded an English-speaking multi-authored article with one non-native co-author to solely consider the authorial identity of the English-speaking community in the field of psychology. Table 2 illustrates the details of our selected samples.

**Table 2:** Details of the samples and journals selected for the analysis.

**Corpus (journals)**	**Impact factor**
Cognition	3.4
Journal of Memory and Language	4.3
Cognitive Psychology	2.6
Brain, Behavior and Immunity	15.1
Journal of Personality	5.4
Iranian Rehabilitation Journal	0.44
Iranian Journal of Clinical Psychology	none cited
Iranian Journal of Social Sciences and Humanities Research	none cited
International Journal of High Risk Behaviors and Addiction	0.43
Iranian Journal of Psychiatry and Behavioral Sciences	0.26

### 3.1 Data analysis

The detection of first-person singular and plural pronouns in each research article in the two corpora was the main focus of the study. To analyze the corpus, we primarily used language corpus tools to detect self-mention markers. To this end, we identified the frequency values of the self-mention markers in the two separate corpora using the 'keyword in context' feature in #Lancsbox (Brezina et al., 2015)—a corpus tool provided by Lancaster University for research purposes—by searching for the self-mention pronouns. Upon software analysis, we double-checked each self-mention marker instances manually to ensure accuracy in our findings. Following this stage, we manually coded each self-mention marker in each RA based on Walková's (2019) three-dimensional taxonomy of self-mention in academic writing. While coding, we excluded verbatim quotations from each text as they were not pertinent to the scope of our study; we solely aimed to explore the article writers' authorial identity, who carried out the research, and not, for instance, their participants' identity in the excerpts. Using SPSS, we also ran a chi-square analysis on our raw data to ascertain whether the differences in each dimension between the two corpora were statistically significant.

The coding of grammatical forms of self-mention markers were clear-cut, and no interpretation was required for their identification. However, in the case of the other two dimensions, namely rhetorical functions and boosting/hedging, we required interpretation beyond the literal meanings of words, as these metadiscoursal features encompass ‘pragmatic effects' (Hyland, 1998). We, therefore, needed to ensure the accuracy of our coding, using inter-coder reliability. For so doing, both researchers independently coded self-mention markers within these two dimensions. The result of Kappa agreement test (κ = 0.93) showed almost perfect agreement (values between 0.81 to 1.00 are considered almost perfect), affirming the acceptable consistency of the data obtained from the coders. Subsequently, any discrepancies were resolved through discussion.

## 4 Results

### 4.1 Frequency of self-mention markers

Initially, the total frequency of self-mention markers in the two corpora were compared, amounting to 633 tokens. A total of 571 tokens were identified in the EC, while the Persian articles contained 62 tokens. The frequency of self-mention within each category was also compared. Table 3 below illustrates the frequency of rhetorical function of self-mentions in the two corpora. As shown in the table, the use of self-mention markers used by English authors were markedly higher than Persian writers.

**Table 3:** Rhetorical function of self-mention in the corpora.

	**Frequency**		**Percent**		**Cumulative Percent**	
	**PC**	**EC**	**PC**	**EC**	**PC**	**EC**
1. Stating contribution	2	8	3.2	1.4	3.2	1.4
2. elaborating argument, presenting opinion, stating knowledge		54		9.5		10.9
3. describing or explaining research decision or procedure	59	454	95.2	79.5	98.4	90.4
4. stating purpose, intention, or focus	1	50	1.6	8.8	100.0	99.1
5. acknowledging others		5		0.9		100.0
Total	62	571	100.0	100.0		

### 4.2 Rhetorical function of self-mention in the corpora

With regard to the rhetorical functions of self-mention markers, it is evident from Table 3 that in both corpora, the third function, “describing/explaining research decision/procedure” had the highest frequency. Similar to English authors (79.5%), Persian authors tended to use most of their self-mention markers for this function (95.2%) but never or rarely used other rhetorical functions (< 5%). The results of Chi-Square test (Table 4) found the difference between the two groups significant (χ^2^ = 12.818, *p* = 0.012 < 0.05).

**Table 4:** Chi-square: comparing rhetorical function in two corpora.

	**Value**	**df**	**Asymptotic significance (2-sided)**
Pearson chi-square	12.818	4	0.012
Likelihood ratio	19.819	4	0.001
Linear-by-linear association	0.205	1	0.651
N of valid cases	633		

The following excerpts illustrate the use of self-mention markers for describing the procedure in the corpora.

**Excerpt 1:** For completeness, **we** also stratified the analysis by time since first vaccine dose by restricting the primary cohort to (v) those who had COVID-19 at least 4 months after their first vaccine dose. **(EC)**

**Excerpt 2: We** provided ten 90-min group MCT sessions to the intervention group, while the control group received no intervention. **(PC)**

Although not significant, the use of self-mention markers for other functions were different in the two corpora. As for stating contribution, authors in both the PC (3.2%) and EC (1.4%) used fewer self-mention markers for this purpose. Authors' use of this function is demonstrated in the following excerpts:

**Excerpt 3:** If true, **our** simulations would demonstrate that parallel retrieval from a record of language is sufficient to produce a behavioral hallmark of syntactic behavior and would add to the growing literature. **(EC)**

**Excerpt 4:** The population includes all real or hypothetical members who are eager to be generalized by findings of **our** research. **(PC)**

With regard to the second function, namely the elaboration of an argument and stating opinion, the native English authors used more markers of this function (9.5%), while Persian non-native English writers used no markers of this type. Excerpt 5 shows the use of this function by native English writers.

**Excerpt 5:** We added two additional items to the PANAS because **we** felt that they were particularly relevant to the graveyard setting (i.e., creeped out and unnerved).

As to the self-mention associated with stating purpose and focus or using signposting language, again the English authors had a greater use of this function (8.8%) compared to their Persian counterparts (1.6%). Moreover, while English authors utilized both “stating the purpose and focus” and “signposting” functionalities, Persian authors solely used the signposting function. The following examples illustrate this rhetorical function in the EC and PC.

**Excerpt 6: We** did not intend to analyze the data for Agreeableness, Neuroticism, and Conscientiousness; these were included at both trait and state level to reduce the possibility of participants discerning the intent of the study, and to increase variation in responses. **(EC) (stating purpose)**

**Excerpt 7:** For a treatment of predictability judgments in situations with varying noise levels, **we** refer the interested reader to our earlier work presented in Schulz et al. (2015). **(EC) (signposting)**

**Excerpt 8:** So many cultural and social factors cause the tendency of youth to drugs which **we** will point most important of them in short. **(PC) (signposting)**

Finally, the use of self-mention in connection with “acknowledging others” was infrequent among both English (0.9%) and Persian authors (0%). The following extract shows the use of such function in the EC.

**Excerpt 9:** To align **our** methods with those used by Prasada and Dillingham (2006), items focused on an individual member of the category rather than the category as a whole (e.g., a single bird vs. birds), and the questions did not include the category label.

### 4.3 Grammatical forms of self-mention in the corpora

The next function to be checked was the grammatical one. Table 5 summarizes the frequency of tokens obtained under this function. As depicted in Table 5, the most frequent form of self-mention in both corpora was subjective pronouns, and the two corpora seemed to have a similar frequency in using both subjective and possessive pronouns.

**Table 5:** Grammatical forms of self-mention in the corpora.

	**Frequency**		**Percent**		**Cumulative Percent**	
	**PC**	**EC**	**PC**	**EC**	**PC**	**EC**
1. Subjective pronouns	54	476	87.1	83.4	87.1	83.4
2. Possessive pronouns	8	89	12.9	15.6	100.0	98.9
3. Other		6		1.1		100.0
Total	62	571	100.0	100.0		

The result of Chi-Square test (χ^2^ = 1.007, *p* = 0.604 > 0.05) in Table 6 also acknowledged that no significant difference exists in the proportion of grammatical forms used in the two corpora.

**Table 6:** Chi-square: comparing grammatical forms in two corpora.

	**Value**	**Df**	**Asymptotic significance (2-sided)**
Pearson chi-square	1.007	2	0.604
Likelihood ratio	1.605	2	0.448
Linear-by-linear association	0.792	1	0.374
*N* of valid cases	633		

As explained, the majority of self-mention markers included subject pronouns in both EC (83.4%) and PC (87.1%). The following excerpts demonstrate the use of subject pronouns in the corpora.

**Excerpt 10:** We first computed descriptive statistics for the personality, psychopathology, and omnibus morphometric variables, as well as bivariate correlations between these variables. **(EC)**

**Excerpt 11: We** selected 467 adolescents with Z scores above 1.5 on the CD scale. **(PC)**

Additionally, the use of possessive pronoun of ‘our' in both EC (15.6%) and PC (12.9%) is shown in the following examples.

**Excerpt 12:** We carried out two sensitivity analyses using alternative classifications of CMV to ensure **our** inferences were consistent across ways of modeling CMV. **(EC)**

**Excerpt 13:** A total of 198 individuals who tested positive for methamphetamine use and met **our** inclusion criteria were included in the study. **(PC)**

Finally, as for the category of ‘other', native English writers made use of six object pronouns, all of which being ‘us', as represented in Excerpt 14.

**Excerpt 14:** This also allowed **us** to assess comprehension for experimental sentences that were followed by semantically-related probe words, which made up half of those trials.

### 4.4 Hedging and boosting of self-mention in the corpora

The hedging and boosting of self-mention was the last category to be compared. As depicted in Table 7, Persian corpus used neither boosting nor hedging in their self-mention markers. In the English corpus, the use of hedging and boosting was also rare. Both corpora had the majority of their self-mention in the neutral form.

**Table 7:** Hedging and boosting of self-mention in the corpora.

	**Frequency**		**Percent**		**Cumulative Percent**	
	**PC**	**EC**	**PC**	**EC**	**PC**	**EC**
1. Boosting		28		4.9		4.9
2. Hedging		6		1.1		6.0
3. Neutral	62	537	100.0	94.0	100.0	100.0
Total	62	571	100.0	100.0		

As shown in Table 8, the results of Chi-Square also showed no significant difference (χ^2^ = 3.79, p =0.151 >0.05) in the proportions.

**Table 8:** Chi-square: comparing hedging and boosting in two corpora.

	**Value**	**Df**	**Asymptotic significance (2-sided)**
Pearson chi-square	3.787	2	0.151
Likelihood ratio	7.009	2	0.030
Linear-by-linear association	3.610	1	0.057
*N* of valid cases	632		

While both groups in the study had almost similar ratios of neutrality, EC writers employed more boosting (4.9%) and hedging (1.1%) devices compared to the PC writers, who used none. Below are examples of self-mention markers with boosting and hedging devices used by the English authors.

**Excerpt 15:** Although not a funneled debriefing protocol **we** were confident from survey responding that mental state attribution was not deemed to be the target of our research. **(Boosting)**

**Excerpt 16: We** felt this was a more appropriate approach given IRT scores provide a more faithful representation of individual response processes when items are categorical (Embretson and Reise, 2000). **(Hedging)**

## 5 Discussion

The study aspired to compare how self-mention markers were used in the method sections of psychology research articles by native English and Persian writers. As the findings of the first question revealed, the use of self-mention markers in the methodology section was a common practice among native English authors. In line with literature (e.g., Martínez, 2005; Khedri, 2016; Jasim Al-Shujairi, 2020; Khedri and Kritsis, 2020; Firdaus et al., 2021), the use of self-mention markers is more common in the methodology section compared to other sections of research articles. The rationale behind this is that authors tend to couple self-mention pronouns with research-and-methodology-related terminologies to not only delineate their particular research methods- as compared to other studies- but also to assert ownership over their work (Khedri and Kritsis, 2020).

Due to the nature of the field of psychology, the prevalent use of self-mention markers, particularly by native English authors, was quite expected in that psychology is inherently a soft science. In line with our findings, Khedri and Kritsis (2020) also concluded that the use of self-mention pronouns is a common practice in psychology research articles. Having compared four disciplines, namely Applied Linguistics, Psychology, Engineering, and Chemistry, they found that disciplinary variations do exist in both the frequency and functionality of self-mention markers; they also concluded that the fields of Applied Linguistics and Psychology contained more authorial references particularly in the methodology sections. Hence, it can be concluded that the use of self-mention markers is more prevalent in soft science disciplines compared to hard sciences due largely to the nature of each field. For one thing, soft sciences often involve more qualitative methods and less straightforward and measurable variables (Hyland, 2001), prompting researchers to accentuate personal engagement, subjective interpretation, and their own roles and perspectives in the research process (Khedri and Kritsis, 2020). Moreover, the subjectivity inherent in data collection methods in soft sciences, such as interviews or observations (Riazi et al., 2023), further underscores the importance of self-mention markers in conveying researchers' involvement and perspectives in such disciplines.

Comparing the use of self-mention markers by native and non-native authors, it was found that native English authors' use of self-mention markers was significantly higher than their Persian counterparts. This greater use of self-mention markers on the part of English writers is in tune with literature (e.g., Walková, 2019; Chen, 2020; Deng and He, 2023) and can be ascribed to their “writer-responsible culture” (McCambridge, 2019) in that they tend to present themselves as the direct contributor to the field. The lower usage of authorial voice on the part of Persian authors could be due to the fact that they believe more objective writing is associated with the avoidance of personal self-mention, while more subjectivity was preferred by their English-speaking counterparts. This notion also pertains to Persian authors' humbleness and a stance of humility in their academic writing which stems from their cultural backgrounds (Shirinbakhsh and Eslami Rasekh, 2013).

The higher number of self-mention markers used by native English writers accounts for their struggle and tendency to get published in international journals. This is because presenting oneself as the primary contributor to the target community will provide scholars with positive reviews from prestigious journals which can be achieved through the use of self-mention markers (Mur Dueñas, 2007). The same assumption can be applied to the current study in which native English writers made use of more self-mention markers to establish their credentials and to manifest their authorial presence in the psychology RAs. This is because using these pronouns allows authors to demonstrate their ownership of the research process, highlight their contribution, and indicate their commitment to the findings. As Hyland (2001) put it, using personal pronouns in this way can also be a marketing strategy for authors, helping them promote their work and establish their reputation in the field.

The authors' decisions to incorporate self-mention are also influenced by the intended audience and the scale of readership (Mur Dueñas, 2007). In this case, RAs by Persian authors predominantly appeared in Iranian journals, indicating a narrower audience compared to international publications. This highlights the impact of cultural and national contexts on authorial presence (Atkinson, 2004). Persian writers, influenced by cultural norms emphasizing positive politeness, may lean toward strategies that enhance solidarity over self-mention markers, unlike their English counterparts who may employ negative politeness strategies, leading to a greater use of self-mention markers. Therefore, it can be concluded that the appropriate level of self-representation in texts especially in academic writings heavily depends on different cultures and traditional viewpoints regarding establishing an authorial presence. Several studies (e.g., Mauranen, 1993; Breivega et al., 2002; Fløttum, 2003; Fløttum et al., 2006; Walková, 2019; Dontcheva-Navratilova, 2023) have also indicated that establishing an authorial-self in academic writing may differ based on the writers' cultural backgrounds.

Furthermore, the study suggests that the status and recognition of scholars within a specific field may also contribute to the frequency of self-mention markers. More professional and distinguished authors of a given community may tend to use more self-mention markers in their RAs, whereas less recognized writers prefer to avoid indicating an authorial presence in their RAs (Mur Dueñas, 2007). The results of the present study is in accordance with this theory. It may be suggested that non-native Persian writers whose RAs were published in Iranian journals may be esteemed as less professional in contrast to English writers whose RAs were published in international and more prestigious journals with a higher impact factor.

The root cause of the less frequent use of self-mention markers in PC may be found in the Iranian culture and educational system (e.g., Sorahi and Shabani, 2016; Zarei and Saadabadi Motlagh, 2019) and the socio-culturally-shaped identities of a given discourse (Ivanic, 1998). In essence, academic writing, as a means of constructing social positioning, reflects diverse senses of self, which is influenced by various discourses and cultures (Hyland, 2002a). Refraining from establishing authority and lack of authorial presence can be attributed to a cultural emphasis on modesty and humility (Chen, 2020; Dontcheva-Navratilova, 2023). Based on such modesty, non-native Persian students are encouraged to adopt a depersonalized writing style from the early stages of learning; this tendency leads to a gradual reduction in the use of self-mention markers, with students increasingly employing passive structures, particularly as they step into the academic world.

Regarding the second research question, self-mention markers were analyzed concerning their threefold functions. As for the first dimension, namely the rhetorical function, it was found that the majority of rhetorical functions in both EC and PC represented the third function, namely the authors' description of the research procedure. This stands to logic in that the main intent of the methods section is to deal with the description and explanation of obtaining data and the procedure of a given study (Swales and Feak, 1994; Peacock, 2011), whereby the researchers can showcase their authorial identity by marketing their own specific procedure and “demarcate the research procedure as their own, laying emphasis on its exclusivity” (Khedri and Kritsis, 2020, p. 10). Our results are in line with the findings of Mur Dueñas (2007) and Hyland (2002a) who analyzed management business and marketing research articles, respectively. They also concluded that the predominant function in research articles across various disciplines is the use of the exclusive pronoun “we” to explain procedures.

In other similar studies, having analyzed the totality of research articles in soft science disciplines, Firdaus et al. (2021), Jasim Al-Shujairi (2020), Kafes (2017), Khedri (2016), Khedri and Kritsis (2020), and Martínez (2005) also found that “explaining the procedure” was the main function of self-mention pronouns through the research articles. For instance, Jasim Al-Shujairi (2020), who compared 32 Non-native Iraqi RAs with 32 ISI ones, found that although non-native authors employed less self-mention markers, the dominant function in both corpora was still the “explaining the procedure”. However, our findings are in contrast with those of Dobakhti and Hassan (2017) who discovered that the most frequent rhetorical function included “stating a goal/purpose”.

As for the function of stating contribution, which is the most powerful rhetorical function when disseminating the results and findings of one's study, both authors in the PC and EC made use of fewer self-mention markers of this function as this function is highly likely to appear in the results section of scholarly papers. Interestingly, even in studies analyzing research papers in their entirety, the function of stating contribution was employed to a limited extent compared to other functions, particularly “explaining the procedure” (e.g., Kafes, 2017; Jasim Al-Shujairi, 2020; Khedri and Kritsis, 2020; Firdaus et al., 2021). Similar findings were also evident in Walková's (2019) analysis of English and non-native Slovak authors and Mur Dueñas (2007) study comparing American and Spanish corpora. Similar to Jasim Al-Shujairi (2020) and Walková's (2019) studies, non-native authors used slightly higher proportion of this function, which could mean that Persian non-native authors may feel more comfortable employing stronger rhetorical functions in smaller academic communities compared to their counterparts, propagating their results to international audiences.

With regard to the function of elaboration of an argument and stating opinion, it was found that native English authors utilized more of these markers (9.5%), whereas Persian non-native English writers used none. The lower proportion of this function is also evident in literature (Walková, 2019; Jasim Al-Shujairi, 2020); however, both studies found that non-native authors had a proportion almost similar to their native counterparts, while in our case, Persian non-native authors used none. In contrast to our findings, Dobakhti and Hassan (2017) found that “elaborating an argument” was one of the most common rhetorical functions. This function- although not common in the methods section- accounts for the authors' cognitive processes (Walková, 2019); therefore, it is not uncommon to be more subjective in the field of psychology, as observed in the native English writers' tendency to incorporate the element of subjectivity in their methods section. However, Persian writers regard the methods section as an objective discourse where no subjective use of authorial identity concerning their cognitive processes is allowed.

As to why in both corpora, the use of “elaborating an argument” is low, it could be due to the fact that authors in the field of psychology are less inclined to assert their authorial identities through cognitive processes, particularly when making claims, elaborating arguments, or suggesting theories. Khedri and Kritsis (2020) also found that authors in the field of engineering and chemistry employed more of this function compared to authors in the field of psychology and applied linguistics. They opined that hard science writers tend to assert their opposing viewpoints through asserting their cognitive processes and elaborating an argument.

Concerning the fourth rhetorical function, namely stating the purpose, intention, or focus, the findings of the current study suggest that there exists a lack of textual signposting language in the academic writing of non-native Persian writers. This finding is in tune with some studies in the literature (e.g., Duszak, 1994; Cmejrková and Daneš, 1997; Jasim Al-Shujairi, 2020), where native speakers leveraged more instances of this function compared to their non-native counterparts. Walková's (2019) finding, however, is in contrast with ours in that she found more self-mention markers of this type on the part of Slovak non-native authors than English authors. According to her findings, the rhetorical function of this type not only mentions the purpose of a given study, but it also regards “forward and backward signposting” in the study (p. 62). In the current study, however, while English authors employed both of these functions, the only use in the PC contained the signposting function.

As to the use of metadiscoursal functions, particularly “forward and backward signposting” in psychology RAs, it has been reported that these are more prevalent in soft science disciplines such as applied linguistics and psychology compared to fields like engineering and chemistry (Khedri and Kritsis, 2020). However, in our study, it was discovered that even native speakers utilized this function to a limited extent, accounting for only 8.8% of usage. One possible explanation for this finding could be the nature of the methods section, which typically includes fewer instances of signposting compared to sections like the Introduction, where authors often incorporate more signposting phrases to guide readers throughout the entire article. Khedri and Kritsis (2020) concluded that this function is commonly found in the Introduction sections of both soft and hard science fields.

The analysis of the last rhetorical function regarding “acknowledging others” revealed that the use of this function was not a common practice in either of the corpora. Our findings align with the findings of Walková (2019) who also found this as the least common function in all the L1 English, L1 Slovak, and L2 English corpora. The primary reason for such paucity is that acknowledging others either concerns leaving unanswered issues to further studies or pertains to the typical disclaimer regarding the research (Walková, 2019), which is often employed toward the end of an article. However, as Walková (2019) stated, this function is not solely confined to the acknowledgment section of the research article and can appear in other sections, including the methodology section.

Although the use of this function was limited in both groups, native English writers did employ it in certain cases. This could be because native writers are more inclined to acknowledge the work of others in the methodology section, enhancing the credibility and validity of their research methods by practicing humility before colleagues (Walková, 2019). Including acknowledgments could also contextualize the methodology and demonstrate the author's awareness of relevant literature. Additionally, native authors may use acknowledgments strategically to highlight limitations, preemptively addressing potential criticisms and demonstrating transparency in their research approach.

The second dimension concerned the grammatical forms. According to the findings, subjective pronouns were the most commonly used self-mentions in both corpora, and there were comparable frequencies in the usage of both subjective and possessive pronouns. Interestingly, all of the pronouns used in both corpora were of plural types. That is, all of the subject pronouns employed in both corpora consisted of “we”, all of the possessive pronouns were “our”, and all of the other pronouns in the EC included “us”. Similar to our study, Khedri (2016) also discovered that ‘we' was mostly employed in the methodology section of research articles. Moreover, Khedri and Kritsis (2020) also discovered that “we-based” pronouns dominated other grammatical forms, with psychology RAs containing the most use of “we-based” pronouns (72.4%) compared to the other three sub-corpora. In the same vein, Dobakhti and Hassan (2017), who analyzed the discussion sections of 150 research papers in Applied Linguistics, also found that first-person plural pronouns such as ‘we' were more common than singular pronouns in both qualitative and quantitative of research articles. Our findings, however, contradicts those of Jasim Al-Shujairi (2020) who found that 66% of the methodology section included ‘I' pronoun, while ‘we' pronoun was the least frequently-used pronoun in this section; he found that it was the “results and discussion” section which entailed the most usage of “we-based” pronouns.

As the majority of RAs written in the field of psychology (including our sample RAs) are multi-authored, it can provide a clear justification as to the absence of subject pronoun “I”, possessive adjective “my”, and the object pronoun “me” in EC and PC. According to Hyland (2001), authors often prefer to maintain a more objective stance, focusing on factual information, thereby reducing the degree of too personal subjectivity. According to him, in order to circumvent the personal interposition and to gain some level of authority, authors are willing to use plural pronouns. This is why most of the writers in EC and PC were inclined to use “we-based” pronouns. This shared tendency among English and Persian writers reflects their preference to take “discourse participant” and “community-self” roles in their RAs (Hyland and Guinda, 2012).

With regard to the last dimension of self-mention in academic articles, it was found that the Persian writers utilized no boosting and hedging in self-mention markers, and such usage was also infrequent in the English corpora. According to the findings, self-mention in both corpora was primarily neutral. In line with the findings of Walková (2019), who also found almost similar ratios between native English and non-native Slovak authors' neutral self-mention markers, the findings of our study also confirmed that the use of hedging and boosting devices in connection with self-mention was not a common practice neither by native English authors nor by their non-native counterparts. This could be due to the fact that the language used in the methods section is mostly declarative. That is, authors mostly seek to describe or explain the procedure through neutral language, avoiding rigid claims (i.e., boosting) or tentativeness (i.e., hedging). Moreover, the dearth of boosting on the part of Persian authors could be indicative of their humbleness in a smaller academic community. Unlike the findings of our study, one study conducted by Karami and Lohran Poor (2020), which compared the hedging and boosting devices in Persian and English psychology books, found that Persian writers used more boosting and fewer hedging devices than their English counterparts. However, they analyzed these devices in the entire texts rather than in connection with self-mention markers.

As to the paucity of the use of hedging and boosting in both groups, it was found that although native English authors used more hedging and boosting, their difference was not significant. The reason why psychology authors in both groups tended to avoid down-toning or asserting language could be attributed to the nature of the field. In a similar vein, Khedri (2016) and Khedri and Kritsis (2020) concluded that these were the hard science authors (i.e., engineering and chemistry authors) who made use of more boosters compared to the applied linguistics and psychology authors. This could be because hard science authors tend to emphasize the significance of their results and conclusions, as their discourse demands more clear-cut findings and decisive conclusions.

## 6 Conclusions and implications

The present contrastive corpus-driven study aimed to examine the use of self-mention markers in the academic writing of two groups of Persian and English authors in the field of psychology. The findings indicated a significant difference in the utilization of self-mention markers in the two corpora. The use of self-mention markers were more prevalent in the English corpus. Generally, English writers tended to establish their authorial stance more firmly than their Iranian counterparts. Moreover, the most common rhetorical function in both corpora pertained to describing and explaining research decision and procedure. With regard to the grammatical forms of self-mention markers, both groups tended to use subject pronouns more frequently. Finally, the use of hedging/boosting devices were almost similar in both groups. Given the differences in the frequency (and some functionalities) of self-mention markers between the two groups, it is safe to claim that Persian writers in the field of psychology tend to adopt a more 'modest', 'objective', and 'detached' stance in their writings. This is largely due to their cultural and educational background, which places emphasis on depersonalization and objectivity in academic writing.

The findings of the present study can be of great help to non-native English writers and scholars to aid them in establishing an authorial voice in their academic writings. Furthermore, gaining knowledge in the area of self-mention markers makes it possible for authors to get out of their silence presence and to take a critical viewpoint in their research articles (Can and Cangir, 2019). The findings of the current study can motivate non-native English writers to take a personal rather than an impersonal stance toward their composition. The results of the current study have implications for both EAP/ESP teachers and students as well. Students and novice writers can take cognizance of the fact that they need to dispel the myth of eschewing self-mention markers in the academic writing simply due to some vague preconceived conventions regarding the impersonality of academic writing. As for teachers, not only should they remind their students of the use of self-mention in their research articles in the field of psychology, but also they need to mention how, where, when, and in what structure to use them. Moreover, it is imperative for materials developers to elucidate the use of self-mention in academic writing, particularly how to employ them judiciously to caution novice researchers against both overuse and underuse of these metadiscoursal features.

One of the limitations of our study included not conducting interviews with native English writers and L1-Persian writers to explore reasons for either using or avoiding self-mention markers in their academic papers. Combining corpus-based studies with interviews from the writers could add invaluable information on the use of metadiscoursal features, making the results more rigorous. Moreover, the present study did not take into account a potential bias in the comparative analysis of the articles regarding the reviewers' comments. In effect, Iranian journals primarily undergo review by local reviewers, while English journals are predominantly reviewed by native speakers; therefore, biases may arise in the revision suggestions regarding the use of personal pronouns. Based on the specific discourse, native speakers might recommend changes to make the language either more objective or, in the current study's case, more subjective, a perspective possibly not shared by Iranian reviewers. Our study, however, did not explicitly address the probability of such biases, and the impact of reviewers' comments or their suggested revisions on the identified self-mention markers is not considered. To enhance the study's robustness, future research could explore the influence of reviewers—for instance through interviews with the writers—on the linguistic features of academic writing in cross-cultural contexts, shedding light on such potential biases.

Additionally, the present study did not examine self-mention on a collocational scale, which can be considered by future researchers to examine the contextual cues and writers' inclination to convey their voice from a collocational viewpoint. Furthermore, as our sample contained 120 articles, further studies can explore more samples to add to the external validity. Moreover, future research might explore self-mention markers across different disciplines, with specific attention to disparities between hard and soft sciences, offering a more nuanced understanding of how self-mention markers function within various academic contexts. Research can also be conducted within a single discipline, comparing different conventions of such metadiscoursal features in different academic discourses, such as master's theses, doctoral dissertations, and journal articles. Finally, future avid researchers can consider the effect of gender on self-mention usage. This could provide valuable insights into male and female authorial identity and how this difference shapes academic writing practices.

## Data availability statement

The raw data supporting the conclusions of this article will be made available by the authors, without undue reservation.

## Author contributions

FM: Writing – original draft. MM: Writing – original draft.
